# The Use of Patient-Specific Induced Pluripotent Stem Cells (iPSCs) to Identify Osteoclast Defects in Rare Genetic Bone Disorders

**DOI:** 10.3390/jcm3041490

**Published:** 2014-12-17

**Authors:** I-Ping Chen

**Affiliations:** Department of Oral Health and Diagnostic Sciences, University of Connecticut Health Center, 263 Farmington Avenue, Farmington, CT 06030, USA; E-Mail: ipchen@uchc.edu; Tel.: +1-860-679-1030

**Keywords:** induced pluripotent stem cells, osteoclast, rare genetic bone disorders

## Abstract

More than 500 rare genetic bone disorders have been described, but for many of them only limited treatment options are available. Challenges for studying these bone diseases come from a lack of suitable animal models and unavailability of skeletal tissues for studies. Effectors for skeletal abnormalities of bone disorders may be abnormal bone formation directed by osteoblasts or anomalous bone resorption by osteoclasts, or both. Patient-specific induced pluripotent stem cells (iPSCs) can be generated from somatic cells of various tissue sources and in theory can be differentiated into any desired cell type. However, successful differentiation of hiPSCs into functional bone cells is still a challenge. Our group focuses on the use of human iPSCs (hiPSCs) to identify osteoclast defects in craniometaphyseal dysplasia. In this review, we describe the impact of stem cell technology on research for better treatment of such disorders, the generation of hiPSCs from patients with rare genetic bone disorders and current protocols for differentiating hiPSCs into osteoclasts.

## 1. Introduction

Studying rare genetic bone disorders is clinically highly relevant. Although individual diseases only affect a small percentage of the population (less than 200,000 people or about 1 in 1500 people in the United States), overall, a large number of people suffer from these skeletal disorders due to their frequency (almost 500 rare genetic bone disorders listed by NIH Office of Rare Disease Research). Many of these diseases become apparent early in life and are present throughout the patient’s entire life. The diverse expressivities of clinical manifestations, from lethality of newborns to mild skeletal abnormalities, make the diagnosis of some of these disorders challenging. Moreover, most of these rare bone diseases are understudied due to the rarity of human specimens and unavailable animal models, and therefore treatment options are often limited or lacking. It is thus important to establish better models for studying such disorders.

Research focusing on genetic disorders of the skeleton is not only beneficial for future treatment of patients, but has significantly contributed to our knowledge on key concepts of bone biology. Rare genetic bone disorders have been linked to abnormal bone development and/or bone remodeling. Pathologically and embryologically these diseases can be subdivided into four major groups: (1) disorders affecting skeletal patterning; (2) disorders of condensation/differentiation of skeletal precursor structures; (3) disorders affecting growth and (4) disorders of bone homeostasis caused by perturbation of interaction between the bone forming osteoblasts and the bone resorbing osteoclasts [1]. Our group has been studying a rare genetic bone disorder, craniometaphyseal dysplasia (CMD) characterized by progressive thickening of craniofacial bones and widening of metaphyses in long bones, utilizing a knock-in mouse model [2]. We have identified defects of osteoblasts and osteoclasts in mice with a CMD mutation [3]. We currently study CMD in a human system using patient-specific induced pluripotent stem cells (hiPSCs) to identify osteoclast defects and we believe this strategy can be applied widely for studying other rare genetic disorders.

In this article, we review some of the rare genetic bone disorders with osteoclast defects, the generation of hiPSCs from patients with rare genetic bone disorders and the protocols for differentiating hiPSC into osteoclasts.

## 2. Rare Genetic Bone Disorders with Osteoclast Defects

Osteoclasts are cells responsible for resorbing bone and work in close concert with osteoblasts to model the skeleton during growth/development and to remodel the bone throughout life. Osteoclasts are derived from the monocyte/macrophage lineage of hematopoietic stem cells and are multinucleated giant cells expressing marker genes, such as tartrate-resistant acid phosphatase (Trap), Cathepsin K, calcitonin receptor, nuclear factor of activated T-cells, cytoplasmic 1 (Nfatc1). Development of osteoclasts (osteoclastogenesis) involves (1) the commitment of hematopoietic stem cells into osteoclast precursors; (2) the fusion of mononucleated osteoclast precursors into multinucleated osteoclast syncytia; (3) the differentiation/maturation to functional osteoclasts. During stage 1, transcription factors PU.1, MITF and c-FOS are important determinants of lineage specification [4,5,6]. In addition, M-CSF signaling is necessary for proliferation and survival of osteoclast progenitors as first became obvious by the osteopetrotic phenotype of mice lacking the *M-CSF* gene (op/op mice) [7]. Interaction of RANKL, a member of TNF family and strongly expressed by osteoblasts, with its receptor RANK on osteoclast progenitors is necessary for the fusion of osteoclast precursors. The activation of RANK/RANKL signaling initiates a cascade of gene expression, including the expression of chemokines such as Mcp-1 to attract RANK^+^ mononuclear osteoclast precursors and molecules important for osteoclast fusion such as Atp6v0d2 and DC-Stamp [8,9]*.* The final step in differentiation to mature and functional osteoclasts involves the formation of a ruffled border and sealing zone. Lack of functional osteoclasts by disruption of these processes or failure of polarization and cytokine organization can lead to osteopetrosis [10].

Many rare genetic bone disorders are partially or primarily caused by osteoclast defects. Dysfunctional osteoclasts can result in too much bone while increased bone resorption can lead to decreased bone mass. Some diseases present a combination of osteosclerosis with osteolytic lesions. Studies on these disorders highlight the important roles of some specific proteins or signaling pathways during osteoclastogenesis.

### 2.1. Diseases of Decreased Osteoclast Resorption

Osteopetrosis: Three main forms of hereditary osteopetrosis are autosomal recessive osteopetrosis (ARO), intermediate autosomal recessive osteopetrosis (IARO) and adult dominant osteopetrosis (ADO), the most severe form being ARO. Mutations causing these diseases generally lead to lack of acid secretion in osteoclasts. ARO presents in infants with severe sclerosis of bone, an increased rate of fracture, extreme reduction of bone marrow space, hepatosplenomegaly, anemia, compression of cranial nerves and growth failure [11]. Infantile malignant osteopetrosis is generally lethal and can only be treated by early bone-marrow transplantation. IARO is a recessive form of osteopetrosis with renal tubular acidosis. The affected protein, carbonic anhydrase type II is highly abundant in osteoclasts, cerebral neurons and in renal intercalated cells. Clinical features of IARO patients include the milder form of osteopetrosis, mental retardation due to cerebral calcification and renal dysfunction [12]. Two distinct types of ADO are known [13]. ADO is characterized by a generalized diffuse osteosclerosis with the most pronounced thickening at the cranial vault in its type I form and the most pronounced abnormalities in vertebrae in type II (Albers Schonberg disease).

Pycnodysostosis: This is an autosomal recessive disorder and can be diagnosed during early infancy. The phenotype is milder than ARO with short stature, recurrent bone fracture, skull deformity and hypoplasia of facial bones, sinuses and clavicles. Long bones are hyperostotic with narrow medullary canals. The calvarium and base of the skull are sclerotic. The genetic defect for pycnodysostosis has been identified in Cathepsin K, a lysosomal cysteine protease required to degrade collagen in resorption lacunae of osteoclasts [14].

### 2.2. Diseases of Increased Osteoclast Resorption

Paget’s disease of bone (PDB): PDB is a late onset bone disease starting at mid-life or later. Genetic predisposition together with environmental risks and other risk factors such as trauma or surgery contribute to its etiology. PDB affects single or multiple locations of the skeleton where focal bone resorption occurs and bone is replaced with soft, fibrous expansile tissue that result in characteristic enlarged and softened bone tissue. Clinical symptoms include bone pain, bone deformity, deafness, pathological fractures and osteoarthritis [15,16]. Although increased osteoclast activity is primarily the cause of PDB, excessive osteoblast activity reflected in elevated serum alkaline phosphatase has been reported [17], which could be a sign of increased bone turnover.

Juvenile PDB (JPDB): Different from Paget’s disease of bone, JPDB usually occurs in infancy or early childhood, characterized by massive thickening of calvaria, widened diaphyses, and deformities of extremities and vertebrae [18]. Autosomal recessive mutations in *TNFRSF11B* result in a less efficient form of osteoprotegerin (OPG) with reduced affinity for RANKL or in a failure to express OPG protein. OPG is a decoy receptor for RANKL, thus regulating osteoclast formation. As a consequence, increased bone resorption coupled with increased bone formation, are seen in JPDB [19,20].

Familial expansile osteolysis (FEO): FEO is an autosomal dominant rare bone disorder characterized by osteolytic lesions in major bones of the appendicular skeleton during early adulthood. It can also result in deafness and premature tooth loss due to abnormalities in the middle ear and jaw [21,22]. Mutations identified in *TNFRSF11A* cause enhanced RANK-mediated nuclear factor-κB (NF-κB) signaling and increased bone remodeling [23].

Expansile skeletal hyperphosphatasia (ESH): ESH is characterized by expanding hyperostotic long bones, early onset deafness, premature tooth loss, episodic hypercalcemia and increased alkaline phosphatase activity; the skull and appendicular skeleton display hyperostosis and/or osteosclerosis [24].

Mutations responsible for these rare disorders affecting osteoclast activity are summarized in Table 1. Research of disorders mentioned above would be greatly enhanced if elaborate models for studying osteoclastogenesis and osteoclast function would be available. Induced pluripotent stem cells (iPSCs) could be one worthwhile avenue to study such disorders in humans. iPS cell approaches have been published for certain bone remodeling disorders described below.

**Table 1:** Mutations in rare genetic bone disorders with osteoclast defects.

Diseases with Decreased Bone Resorption				
Disease	OMIM	Gene affected	Protein affected	Reference(s)
ARO	259,700	*TCIRG1*	α3 Subunit of vacuolar proton pump H^+^ ATPase	[1,25]
ARO	259,700	*CLCN7*	Chloride channel	[26]
ARO	259,700	*OSTM1*	GL	[27]
IARO	259,730	*CAII*	Carbonic anhydrase II	[12]
ADOI	166,600	*Lrp5*	Lrp5	[28]
ADOII	166,600	*CLCN7*	Chloride channel	[29]
Pycnodysostosis	265,800	*CTSK*	Cathepsin K	[14]
PDB	6,002,080	*SQSTM1*	P62	[30,31]
JPDB	239,000	*TNFRSF11B*	Osteoprotegerin (OPG)	[19]
FEO	174,810	*TNFRSF11A*	RANK	[23]
ESH	N/A	*TNFRSF11A*	RANK	[32]

ARO: autosomal recessive osteopetrosis; IARO: intermediate autosomal recessive osteopetrosis; ADOI: adult dominant osteopetrosis, type I; ADOII: adult dominant osteopetrosis type II; PDB: Paget’s disease of bone; JPDB: Juvenile Paget’s disease of bone; FEO: familial expansile osteolysis; ESH: expansile skeletal hyperphosphatasia; OMIM: Online Mendelian Inheritance in Man; GL: Grey lethal; N/A: not available.

## 3. Generation of hiPSCs from Rare Genetic Bone Disorders

hiPSCs, similar to human embryonic stem cells (hESCs), have the ability to self-renew indefinitely and in theory differentiate to any cell type when induced under appropriate conditions. The advances in hiPSCs technology opened new opportunities for medical research in disease modeling, drug screening, gene therapy and genome editing [33,34,35]. Generation of hiPSCs can, for example, be achieved by introduction of reprogramming factors, *OCT3/4*, *SOX2*, *KLF4* and *c-MYC* or *OCT3/4*, *SOX2*, *NANOG*, and *LIN28* [36,37,38]. Methods successfully delivering these reprogramming factors into somatic cells include transduction with retroviruses, lentiviruses, adenoviruses, piggyBac transposons, episomal vectors, RNA, or protein [37,39,40,41,42,43,44]. Many types of somatic cells have been reprogrammed into hiPSCs, including fibroblasts, keratinocytes, peripheral blood mononuclear cells, cord blood cells, T cells, dental pulp stem cells, dermal papilla cells from hair follicles and urinary cells [37,45,46,47,48,49]. Patient-specific hiPSCs provide unique opportunities for researchers to dissect the pathogeneses and identify potential treatment strategies for the rare genetic bone disorders by providing a virtually unlimited source of cells carrying the disease-causing mutations. hiPSCs can be differentiated into functional cells of interest in the skeletal system, including osteoclasts. hiPSC disease modeling has been established for several non-skeletal disorders including type I and type II diabetes, muscular dystrophy, amyotrophic lateral sclerosis, Parkinson’s disease, glioblastoma, familial platelet disorder with predisposition to acute myeloid leukemia (FPD) [36,50,51,52,53,54,55]. There have been only few attempts to establish hiPSCs from patients with rare genetic bone disorders (see also Table 2).

**Table 2:** iPSCs generated from patients with rare genetic bone disorders.

Disease	Source of Somatic Cells	Method	Reprogramming Factors	Patient Numbers	Reference
OI	MSC derived from bone fragments	(1) lentivirus	(1) *OCT4*, *SOX2*, *LIN28* or *NANOG*	6	[56]
		(2) floxed, polycystronic foamy virus	(2) *OCT4*, *SOX2*, *KLF4* and *c-MYC*		
CMD	5–7 mL peripheral blood	Sendai virus	*OCT3/4*, *SOX2*, *KLF4* and *c-MYC*	8	[57]
FOP	Dermal fibroblasts	(1) retrovirus	(1) *OCT4*, *SOX2*, *KLF4* and *c-MYC*	5	[58]
		(2) episomal vectors	(2) *SOX2*, *KLF4*, *OCT4*, *L-MYC*, *LIN28*, *p53*		
MFS	Dermal fibroblasts	retrovirus	*OCT4*, *SOX2*, *KLF4* and *c-MYC*	2	[59]

OI: osteogenesis imperfecta; CMD: craniometaphyseal dysplasia; FOP: Fibrodysplasia ossificans progressiva; MFS: Marfan syndrome; MSC: mesenchymal stem cells.

Osteogenesis Imperfecta (OI): OI, also known as brittle bone disease, is characterized by brittle bones that are prone to fracture and are caused by mutations in the *COL1A1* or *COL1A2* genes in the majority of cases. Misfolded collagen overwhelms the protein degradation machinery of cells and leads to abnormal bone matrix deposition by osteoblasts. 8 Types of OI have been identified. There is currently no cure for OI and treatment focuses on the prevention of fractures and the maintenance of mobility [60]. Deyle *et al.* established mesenchymal cell cultures from discarded bone fragments of OI patients undergoing surgery and further inactivated mutant collagen genes by adeno-associated virus (AAV)-mediated gene targeting, thus preventing the expression of misfolded collagen protein. OI and gene-targeted OI mesenchymal cells have been reprogrammed to hiPSCs [56].

Craniometaphyseal dysplasia (CMD): CMD is characterized by progressive hyperostosis of craniofacial bones and widened metaphyses of long bones. Patients often suffer from blindness, deafness, facial paralysis and severe headache due to hyperostosis and compression of the brain and nerves. Mutations for the autosomal dominant form of CMD have been identified in of progressive ankylosis (*ANKH*) gene and for a recessive form in Connexin 43 (*Cx43*) [61,62,63]. Our group identified dysfunctional osteoclasts in knock-in (KI) mice carrying a Phe377del mutation and in human osteoclast cultures [2,3]. The increased bone mass phenotype in CMD mice (*Ank*^KI/KI^ mice) can partially be rescued by bone marrow transplantation. We have established a simple and efficient method to generate integration-free hiPSCs from peripheral blood of CMD patients and healthy controls using the Sendai virus, a cytoplasmic RNA viral vector [57], that can easily be removed from cells after reprogramming to iPSCs.

Fibrodysplasia ossificans progressiva (FOP): FOP is a rare genetic disorder caused by hyperactive mutations in the bone morphogenetic protein (BMP) type I receptor ACVR1 [64]. It is characterized by progressive ossification of soft tissues. The mechanism of heterotopic ossification is endochondral bone formation which involves pre-cartilaginous, fibro-proliferative and mineralization stages. Matsumoto *et al.* generated hiPSCs from skin fibroblasts of FOP patients and controls and showed increased *in vitro* chondrogenic differentiation and mineralization in FOP hiPSCs compared to wild type hiPSCs [58].

Marfan syndrome (MFS): MFS is a life-threatening, autosomal dominant disease with mutations identified in *FIBRILLIN-1* (*FBN1*) [65]. It is a disorder of fibrous connective tissue involving three systems: skeletal, cardiovascular and ocular. Skeletal features include long limbs and digits, deformities of vertebrae (scoliosis, thoracic lordosis) and anterior chest, increased height, and mild to moderate joint laxity. Quarto *et al.* generated hiPSCs from MFS patients and studied the pathogenic skeletogenesis *in vitro* [59]. They show that MFS-hiPSC faithfully represent the impaired osteogenic differentiation as a consequence of activation of TGF-β signaling and revealed a crosstalk between BMP and TGF-β signaling in MFS [66].

## 4. Differentiating hiPSCs into Osteoclasts

### 4.1. Differentiating Mouse Embryonic Stem Cells (mESCs) into Osteoclasts

Several studies have reported the generation of osteoclasts from mESC lines by culturing mESCs directly on a culture plate or by co-culturing mESCs with mouse bone marrow-derived stromal cells (ST2) or with the newborn calvaria-derived stromal cell line (OP9) from mice deficient in macrophage colony-stimulating factor (M-CSF) or through EB formation (for details see Table 3). Information gained from these mouse ESCs/iPSCs studies provided the fundamentals for establishing methods to generate osteoclasts from human ESCs and iPSCs.

**Table 3:** Protocols for differentiating mESCs/iPSCs to osteoclasts.

Methods	Mouse ESC Lines	Factors Added in OC Medium	Results	Reference	Lessons Learned
mESCs on 24-well plates	D3, J1	hM-CSF, hRANKL, A.A, VitD_3_, Dexa	TRAP+ cells (day 14)	[67]	A.A. increased total cell recovery and OC precursors through increasing Flk-1-positive cells when added during the initial 4 days.
Co-culture 1-step, 2-step, 3-step	D3	hM-CSF (for OP9 coculture) VitD_3_, Dexa	TRAP+ cells (day 11–16)	[68]	ST2 supported osteoclastogenesis more efficiently than OP9. C-fms signaling is required for OC development from mESCs.
Co-culture 1-step, 2-step, 3-step	CCE, D3, J1, CJ7	hM-CSF (for CFU assay) VitD_3_, Dexa	TRAP+ cells (day 11–16)	[69]	SCL is indispensable for osteoclastogenesis. GATA-2 is required for osteoclastogenesis at early but not terminal differentiation stage.
Co-culture 1-step	D3	VitD_3_, Dexa, hRANKL, hM-CSF (for some exp.)	TRAP+ cells c-Kit, c-fms, β2-integrin, CD31 expression (day 3–17)	[70]	Temporal expression of markers: c-Kit → β2-integrin → c-fms, TRAP. Exogenous hM-CSF and hRANKL promote osteoclastogenesis. Continuous hM-CSF can reduce number of TRAP+ cells.
Co-culture 1-step, 2-step, 3-step	D3, CCE	VitD_3_, Dexa	TRAP+ cells	[71]	Blocking VEGFR-mediated signaling is inhibitory to OC development.
EB	mESCs	mM-CSF, mRANKL	TRAP+ (≥3 nuclei) (day 13)	[72]	Efficiency of OC generation: 3-step coculture > EB method > 1-step coculture.
EB, monolayer culture	J1, miPSCs (38c2, 20D17)	M-CSF, RANKL	TRAP+ (≥3 nuclei) (day 19)	[73]	A new *in vitro* culture method to differentiate mES/iPSCs into osteoclasts.

A.A: 50 μg/mL ascorbic acid; VitD_3_: 10^−8^ M 1α,25-dihydroxyvitamin D_3_; Dexa: 10^−7^ M dexamethasone; 1-step: mESCs are cocultured with ST2 cells for 10 days; 2-step: mESCs are cocultured with OP9 cells for 5 days and transferred onto ST2 cells for 6 days; 3-step: mESCs are cocultured with OP9 for 5 days, transferred to new OP9 for further 5 days, transferred onto ST2 cells for 6 more days; OC: osteoclasts; Exp.: experiments.

### 4.2. Commitment of Human ESCs/hiPSCs into Hematopoietic Lineages/OC Precursors

Consistent and adequate hematopoietic differentiation of hiPSCs is a prerequisite step for differentiating hiPSCs into osteoclasts. Hematopoiesis during embryogenesis starts with the formation of the primitive streak, mesoderm differentiation and hematopoietic specification. *In vitro* studies have shown that hiPSCs can be differentiated into different hematopoietic lineages through similar processes. Inducing hematopoiesis from human embryonic stem cells (hESCs) or hiPSCs has been reported by several extensive studies using three systems: (1) differentiation by coculturing hESC/hiPSCs with stromal cells; (2) differentiation through formation of embryoid bodies (EB), which can be differentiated into the three germ layers including mesoderm; (3) differentiation by monolayer culture of hESCs/hiPSCs on extracellular matrix protein coated-plates, such as collagen IV. We summarize culture conditions of these protocols in Table 4.

There are advantages and disadvantages inherent to these protocols. Differentiation efficiency of co-culture methods relies largely on optimized cell densities of hESCs/hiPSCs and the mouse stromal cell lines. It can be challenging to have both cell culture systems ready for co-culture at the same time. On the other hand, co-culture method requires less hematopoietic cytokines and is therefore relatively inexpensive compared to protocols involving EB and monolayer cultures. Concentrations of cytokines used are critical in those cultures. Stimulatory or inhibitory effects of cytokines towards hematopoiesis can be observed depending on the concentrations used. It is difficult to directly compare the lengths of cell culture time or hematopoietic differentiation efficiencies among the approaches described in Table 4. Variability of culture conditions used for the experiments among these protocols is discussed below.

### 4.3. Marker Genes for Mesodermal Formation and Hematopoietic Differentiation

Marker genes for mesodermal and hematopoietic lineages have been used to determine the efficiency of hematopoietic differentiation protocols during *in vitro* differentiation. Temporal expression patterns of certain marker genes are reliable indicators of mesoderm and hematopoietic differentiation from undifferentiated hESCs or hiPSCs. Early mesoderm formation is indicated by the expression of marker genes such as *Brachyury* (*T*), *Mix paired-like homeobox* (*Mixl1*), *Goosecoid homeobox* (*GSC*), and silencing of the pluripotency genes [74,75]. While kinase insert domain receptor (a type III receptor tyrosine kinase, *KDR*), also known as endothelial growth factor receptor 2 (*VEGFR-2*) or fetal liver kinase 1 (*Flk1*), is already detected in hESC/hiPSCs, its expression increases during the transition from mesoderm to hematopoietic lineage [76]. In addition, transcription factors stem cell leukemia (*Scl/Tal-1*), runt-related transcription factor 1 (*Runx1*), globin transcription factor 1 (*GATA-1*) and *GATA-2* play important roles in hematopoietic commitment during embryogenesis [77,78,79]. Combinations of surface marker expression are used to detect the hematopoietic stem cells (HSC) or the maturation of HSCs into specific hematopoietic cells. CD34, CD31 and VE-cadherin are expressed in early hematopoietic cells and vascular associated tissues [76]. CD45 is a pan-leukocyte marker [80]. Lin^−^CD34^+^CD43^+^CD45^+^ cells represent a population of enriched myeloid progenitors [81].

**Table 4:** Protocols for hematopoietic differentiation from hiPSCs.

Methods	hES/iPSCs & Medium	Differentiation medium	Results	Reference	Protocol Time Line
Monolayer	KhES-1, KhES-3, 201B7, 253G4 mTeSR1, Stemline II	Stemline II + ITS	T + Mixl1+ cells (d4) KDR+ CD34^+^CD45^−^ cells (d6) 36% CD235a^+^; 53% CD45^+^ (d30)	[82]	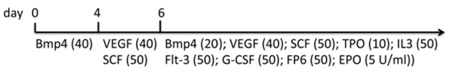
Monolayer (Collagen IV)	WA01 hiPSCs Matrigel/mTeSR1	IMDM, BIT, MTG, NEAA, l-glu	95% CD43^+^, 53% CD34^+^, 59% CD41a^+^, 60% CD235a^+^, 35% CD45^+^ (d14)	[83]	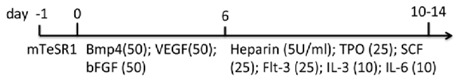
EB monolayer (gelatin)	hiPSCs MEF/hESC medium	EB^1^ medium/monocyte differentiation medium	90% CD14^+^ (d15 of attached, flatten EBs on gelatin plates)	[84]	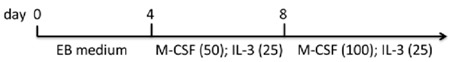
EB	WA01, H9 Matrigel/condition medium	Knockout DMEM, FBS, NEAA, l-glu, ME	9.3% CD45^+^ (d15)	[85]	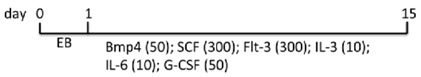
EB	WA01, ES02 MEF/hESC medium	StemPro-34 + MTG + l-glu + A.A.	Mesoderm induction and hemangioblast development (d1-8), increased *T* (d3), CD34, SCL (d5), CD117^+^CD31^+^ (d8)	[76]	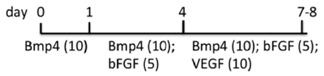
EB	hFib2-iPS5 MEF/hESC medium	EB^2^ medium	29% CD34^+^, 27% CD45^+^, 16% CD34^+^CD45^+^ (d17)	[86]	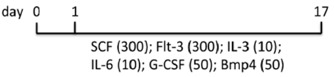
EB	WA01, ES02, MSC-iPS1 matrigel/hESC medium	StemPro34 + MTG + l-glu + A.A.	15%–59% CD45^+^ (d14); 38%–72% CD45^+^ (d22)	[87]	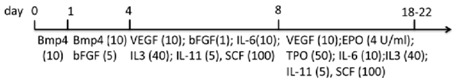
EB	hiPSCs Matrigel/hESC medium	StemPro-34, l-glu, A.A., transferrin, MTG	Myeloid, erythroid, megakaryocytic cells released into the medium (d14)	[88]	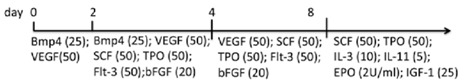
Co-culture (S17/C166)	H1, H1.1, H9.2 MEF/hESC medium	DMEM, FBS, l-glu, ME, NEAA	1%–2% CD34^+^CD38^−^ (d17)	[89]	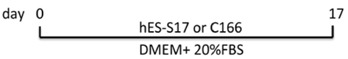
Co-culture (AM-20, UG26, IL08, AGM, FL)	H1, H9, hES-NCL1 MEF/hESC medium	Knockout DMEM, FCS, ME, l-glu, NEAA, antibiotics	16% CD34^+^, 5%CD45^+^, 8% CD31^+^, 6% CD34^+^CD31^+^ (d18)	[90]	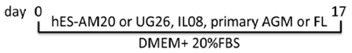
Co-culture (OP9)	WA01, WA09, iPS-1, iPSCs (SK46)-M-4-10 MEF/hESC medium	α-MEM, FBS, MTG	9.8% CD43^+^, 14% CD45^+^ (d9) 94% CD43^+^, 78% CD45^+^ (d11) 98% CD43^+^, 97% CD45^+^ (d17)	[81]	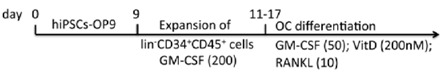

hESC medium: DMEM/F12, 15% KnockOut SR replacer, 2 mM l-glutamine, 0.1 mM β-mercaptoethanol, bFGF. FP6: complex of IL-6 and IL-6 receptor; A.A.: ascorbic acid (50 μg/mL); MTG: 4 × 10^−4^ M monothioglycerol; l-glu: 2 mM glutamine; EB^1^ medium: DMEM-F12, 20% Knockout Serum Replacement, 0.1 mM nonessential amino acids (NEAA), 0.1 mM β-mercaptoethanol (ME), 1 mM l-glutamine; EB^2^ medium: Knockout Dulbecco modified Eagle medium, 20% fetal calf serum, 0.1 mM nonessential amino acids (NEAA), 0.1 mM β-mercaptoethanol (ME), 1 mM l-glutamine, 50 μg/mL ascorbic acid, 201 μg/mL human holo-transferrin; S17: murine bone marrow cell line; C166: murine yolk sac endothelial cell lineAM20, UG26; EL08: stromal cell lines; AGM or FL: primary stromal cells from aorta-gonad-mesonephros (AGM) or fetal liver (FL); BIT: bovin serum albumin, human recombinant insulin, human transferrin; cytokine concentration (ng/mL).

### 4.4. Factors and Cytokines to Promote Hematopoiesis

Defined factors/cytokines can be added to support hematopoietic cell proliferation or differentiation under controlled conditions. BMP4 alone can induce primitive streak and early hematopoietic gene expression, including *Mixl1*, *Brachyury*, *Goosecoid*, *KDR*, *Runx1* and *Gata2* while BMP4 together with VEGF can increase expression of *Scl* and *CD34* [91]. The addition of FGF2 during hematopoietic differentiation of hESCs increases total cell number by improving cell proliferation but not cell survival [91]. A mixture of cytokines including stem cell factor (SCF), fms-like tyrosine kinase receptor-3 ligand (Flt-3), interleukin-3 (IL-3), interleukin-6 (IL-6) and granulocyte colony-stimulating factor (G-CSF) and thrombopoietin (TPO) is commonly used in hematopoietic differentiation protocols for hESCs. These cytokines have been shown to play important roles in maintenance of human hematopoietic cells [92,93]. Addition of SCF in the presence of BMP4, VEGF and FGF can significantly increase the yields of hematopoietic progenitors and mature cells *in vitro* [91]. When added to IL-3 and GM-CSF, SCF has profound effects on *in vitro* proliferation of primitive hematopoietic progenitors [94].

### 4.5. Variability among Hematopoietic Differentiation Protocols

Some studies showed variable efficiencies for deriving hematopoietic cell populations from hESCs/hiPSCs. Many factors contribute to this variability including the somatic cell type used for hiPSC reprogramming; the method of deriving hiPSCs; incomplete removal of reprogramming transgenes in hiPSCs; the culture conditions for maintaining hiPSCs; the type of differentiating medium, growth factors and hematopoietic cytokines added in the differentiation protocol; the dosage of cytokines to promote hematopoiesis; the oxygen level of cultures (normal oxygen or hypoxia); and the size of EBs or the densities of stromal cell lines [95]. It has been suggested that epigenetic memory exists in hiPSCs and the differentiation phenotype may be influenced by their cells of origin [96]. Transgenes remaining in hiPSCs can have a negative impact on hiPSC differentiation [97]. Multiple concentrations of BMP4 (5, 10, 25, 50 ng/mL) were tested and showed no differences in promoting CD34^+^CD45^+^ populations [85] while Pick *et al.* showed BMP4 increases primitive streak and hematopoietic gene expressions in a dose-dependent manner [91]. It is therefore important to develop a method that eliminates as many variable factors as possible and that aims to obtain hematopoietic cells from hiPSCs by a reproducible and efficient protocol.

### 4.6. Differentiating hiPSCs-Derived Osteoclast Progenitors into Osteoclasts

Two publications describe the successful differentiation of hiPSCs to hematopoietic cell stage and further to mature OC progenitors and into functional osteoclasts. Choi *et al.* cultured a Lin^−^CD34^+^CD43^+^Cd45^+^ population in the presence of GM-CSF and vitamin D3 on poly (2-hydroxyethyl methacrylate)-coated plates for 2 days to expand osteoclast progenitors and then induced osteoclast maturation in cultures with α-MEM, 10% FBS, and MTG solution supplemented with GM-CSF (50 ng/mL), Vitamin D3 (200 nM) and RANKL (10 ng/mL) [81]. Grigoriadis *et al.* cultured myeloid precursors derived from hiPSCs in IMDM containing 10% FCS, M-CSF (10 ng/mL) and RANKL (10 ng/mL). Osteoclasts were defined as multinucleated cells (≥3 nuclei) by TRAP positive staining and the capability of resorbing bone/dentin chips. Expressions of OC marker genes such as Cathepsin K, calcitonin receptor, NFATc1 are increased in these functional OCs [87].

Studies summarized in Table 3 and Table 4 demonstrate that osteoclasts can be generated from both, human and mouse ES/iPSCs. In general, differentiation of osteoclasts from hESCs/hiPSCs systems is more challenging than differentiation from mouse ESCs/iPSCs. Differentiation of hESCs/hiPSCs requires more hematopoietic cytokines, longer culture periods and generally results in less efficient osteoclast formation. However, the mechanisms behind differences between human and mouse osteoclastogenesis are still unclear.

### 4.7. Strategies of using hiPSC-Osteoclasts to Study Rare Genetic Bone Diseases

hiPSC technology in general enables researchers to reprogram somatic cells into an ES-like state followed by differentiation into desired cell type. While human osteoclasts can be differentiated directly from peripheral blood, the big advantage of hiPSC technology is that osteoclasts can be generated without repeated sampling of patients. hiPSCs provide a virtually unlimited cell source to study molecular mechanisms of osteoclastogenesis with the potential to develop therapies, which is especially important when studying rare genetic bone diseases. Because of potential species-specific differences, studying abnormal osteoclastogenesis in the human system may be closer to clinical reality than using animal models.

**Figure 1:**
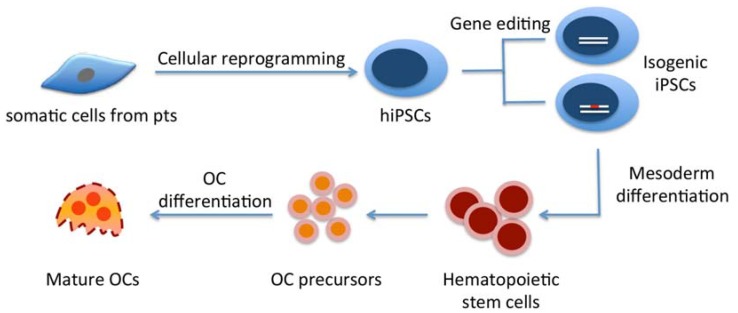
Summary of generating osteoclasts from human iPSCs.

A preferred strategy for studying defective osteoclastogenesis in rare genetic bone disorders using patient-specific hiPSCs is summarized in Figure 1. One challenge of hiPSC disease modeling *in vitro* is the lack of genetically matched controls. Using healthy subjects as controls may not be the best solution as individual hESCs and hiPSC lines differentiate to specific cell populations with variable efficiency because of biological variability [98]. Distinguishing mutation-relevant disease phenotypes from genetic/epigenetic variations becomes easier in isogenic hiPSCs, which only differ at disease-causing mutations. Correction or introduction of specific mutations into a cell can be achieved by genome editing using zinc-finger nucleases (ZFNs), transcription activator-like effector nucleases (TALENs) or the clustered regulatory interspaced short palindromic repeats/Cas9 system (CRISPR) [99,100,101,102]. Using step-wise OC differentiation protocols for differentiating hESCs or hiPSCs may allow researchers to identify which step of osteoclastogenesis is disrupted by a disease causing mutation. Analysis tools are available to study each step (lineage determination of precursors, precursor proliferation, fusion to multinucleated syncytia, maturation to functional osteoclasts) such as expression of marker genes, numbers of TRAP^+^ mono/multinucleated cells, resorption efficiency, live-image migration assays and nuclear localization of NFATc1. Therapeutic strategies can be investigated once the pathologic mechanisms are understood.

## 5. Conclusions

Osteoclast defects are involved in many rare genetic bone diseases as well as in some common bone diseases such as osteoporosis. Although human OC can be cultured from peripheral blood, being able to differentiate OCs from hiPSCs has at least two additional advantages: (1) eliminating the need to repeatedly obtain blood from study subjects; (2) serving as an *in vitro* model for studying hematopoiesis during embryogenesis. Lessons learned from embryology and differentiation studies are expected to improve protocols for consistent and efficient differentiation of hiPSCs into hematopoietic cells and further into osteoclasts. Similar concepts can be applied to differentiate hiPSCs to other bone cells such as osteoblasts. We believe this model will have great impact on a better understanding of bone diseases and to establish the bases for potential therapies.
